# Supplementary material for: An Introduction to Programming for Bioscientists: A Python-Based Primer
Source: PLoS Comput Biol. 2016 Jun 7;12(6):e1004867. doi: 10.1371/journal.pcbi.1004867 (PMC4896647; doi:10.1371/journal.pcbi.1004867)
Supplement: S1 Text — This suite of 19 Supplemental Chapters covers the essentials of programming. The Chapters are written in Python and guide the reader through the core concepts of programming, via numerous examples and explanations. The most recent versions of all materials are maintained at http://p4b.muralab.org. For purposes of self-study, solutions to the in-text exercises are also included. (ZIP) [file pcbi.1004867.s001.zip › zellegraphics.pdf]

Graphics Module Reference

John M. Zelle

Version 4.1, Fall 2010

1 Overview

The package graphics.py is a simple object oriented graphics library designed to make it very easy for novice programmers to experiment with computer graphics in an object oriented fashion. It was written by John Zelle for use with the book "Python Programming: An Introduction to Computer Science" (Franklin, Beedle & Associates). The most recent version of the library can obtained at http://mcsp.wartburg.edu/zelle/python. This document is a reference to the functionality provided in the library. See the comments in the file for installation instructions.

There are two kinds of objects in the library. The GraphWin class implements a window where drawing can be done, and various GraphicsObjects are provided that can be drawn into a GraphWin. As a simple example, here is a complete program to draw a circle of radius 10 centered in a 100x100 window:

```
from graphics import *

def main():
    win = GraphWin("My Circle", 100, 100)
    c = Circle(Point(50,50), 10)
    c.draw(win)
    win.getMouse() # pause for click in window
    win.close()

main()
```

GraphWin objects support coordinate transformation through the setCoords method and pointer-based input through getMouse. The library provides the following graphical objects: Point, Line, Circle, Oval, Rectangle, Polygon, Text, Entry (for text-based input), and Image. Various attributes of graphical objects can be set such as outline-color, fill-color and line-width. Graphical objects also support moving and hiding for animation effects. An additional class, Pixmap, is provided for simple image manipulation tasks.

2 GraphWin Objects

A `GraphWin` object represents a window on the screen where graphical images may be drawn. A program may define any number of `GraphWins`. A `GraphWin` understands the following methods:

`GraphWin(title, width, height)` Constructs a new graphics window for drawing on the screen. The parameters are optional, the default title is "Graphics Window," and the default size is 200 x 200.

`plot(x, y, color)` Draws the pixel at (x, y) in the window. Color is optional, black is the default.

`plotPixel(x, y, Color)` Draws the pixel at the "raw" position (x, y) ignoring any coordinate transformations set up by `setCoords`.

`setBackground(color)` Sets the window background to the given color. The initial background is gray. See Section 5.8.5 for information on specifying colors.

`close()` Closes the on-screen window.

`getMouse()` Pauses for the user to click a mouse in the window and returns where the mouse was clicked as a `Point` object.

`checkMouse()` Similar to `getMouse`, but does not pause for a user click. Returns the latest point where the mouse was clicked or `None` if the window as not been clicked since the previous call to `checkMouse` or `getMouse`. This is particularly useful for controlling simple animation loops.

`setCoords(xll, yll, xur, yur)` Sets the coordinate system of the window. The lower-left corner is (xll, yll) and the upper-right corner is (xur, yur). All subsequent drawing will be done with respect to the altered coordinate system (except for `plotPixel`).

3 Graphics Objects

The module provides the following classes of drawable objects: `Point`, `Line`, `Circle`, `Oval`, `Rectangle`, `Polygon`, and `Text`. All objects are initially created unfilled with a black outline. All graphics objects support the following generic set of methods:

`setFill(color)` Sets the interior of the object to the given color.

`setOutline(color)` Sets the outline of the object to the given color.

`setWidth(pixels)` Sets the width of the outline of the object to this many pixels. (Does not work for `Point`.)

`draw(aGraphWin)` Draws the object into the given `GraphWin`.

`undraw()` Undraws the object from a graphics window. This produces an error if the object is not currently drawn.

`move(dx,dy)` Moves the object `dx` units in the x direction and `dy` units in the y direction. If the object is currently drawn, the image is adjusted to the new position.

`clone()` Returns a duplicate of the object. Clones are always created in an undrawn state. Other than that, they are identical to the cloned object.

3.1 Point Methods

`Point(x,y)` Constructs a point having the given coordinates.

`getX()` Returns the x coordinate of a point.

`getY()` Returns the y coordinate of a point.

3.2 Line Methods

`Line(point1, point2)` Constructs a line segment from `point1` to `point2`.

`setArrow(string)` Sets the arrowhead status of a line. Arrows may be drawn at either the first point, the last point, or both. Possible values of `string` are `'first'`, `'last'`, `'both'`, and `'none'`. The default setting is `'none'`.

`getCenter()` Returns a clone of the midpoint of the line segment.

`getP1(), getP2()` Returns a clone of the corresponding endpoint of the segment.

3.3 Circle Methods

`Circle(centerPoint, radius)` Constructs a circle with given center point and radius.

`getCenter()` Returns a clone of the center point of the circle.

`getRadius()` Returns the radius of the circle.

`getP1(), getP2()` Returns a clone of the corresponding corner of the circle's bounding box. These are opposite corner points of a square that circumscribes the circle.

3.4 Rectangle Methods

`Rectangle(point1, point2)` Constructs a rectangle having opposite corners at `point1` and `point2`.

`getCenter()` Returns a clone of the center point of the rectangle.

`getP1(), getP2()` Returns a clone of corner points originally used to construct the rectangle.

3.5 Oval Methods

`Oval(point1, point2)` Constructs an oval in the bounding box determined by `point1` and `point2`.

`getCenter()` Returns a clone of the point at the center of the oval.

`getP1(), getP2()` Returns a clone of the corresponding point used to construct the oval.

3.6 Polygon Methods

`Polygon(point1, point2, point3, ...)` Constructs a polygon having the given points as vertices. Also accepts a single parameter that is a list of the vertices.

`getPoints()` Returns a list containing clones of the points used to construct the polygon.

3.7 Text Methods

`Text(anchorPoint, string)` Constructs a text object that displays the given `string` centered at `anchorPoint`. The text is displayed horizontally.

`setText(string)` Sets the text of the object to `string`.

`getText()` Returns the current string.

`getAnchor()` Returns a clone of the anchor point.

`setFace(family)` Changes the font face to the given `family`. Possible values are `'helvetica'`, `'courier'`, `'times roman'`, and `'arial'`.

`setSize(point)` Changes the font size to the given `point` size. Sizes from 5 to 36 points are legal.

`setStyle(style)` Changes font to the given `style`. Possible values are: `'normal'`, `'bold'`, `'italic'`, and `'bold italic'`.

`setTextColor(color)` Sets the color of the text to `color`. Note: `setFill` has the same effect.

4 Entry Objects

Objects of type `Entry` are displayed as text entry boxes that can be edited by the user of the program. `Entry` objects support the generic graphics methods `move()`, `draw(graphwin)`, `undraw()`, `setFill(color)`, and `clone()`. The `Entry` specific methods are given below.

`Entry(centerPoint, width)` Constructs an `Entry` having the given center point and `width`. The `width` is specified in number of characters of text that can be displayed.

`getAnchor()` Returns a clone of the point where the entry box is centered.

`getText()` Returns the string of text that is currently in the entry box.

`setText(string)` Sets the text in the entry box to the given string. Changes the font face to the given `family`. Possible values are: `'helvetica'`, `'courier'`, `'times roman'`, and `'arial'`.

`setSize(point)` Changes the font size to the given `point` size. Sizes from 5 to 36 points are legal.

`setStyle(style)` Changes font to the given `style`. Possible values are: `'normal'`, `'bold'`, `'italic'`, and `'bold italic'`.

`setTextColor(color)` Sets the color of the text to `color`.

5 Displaying Images

The graphics module also provides minimal support for displaying and manipulating images in a `GraphWin`. Most platforms will support at least PPM and GIF images. Display is done with an `Image` object. Images support the generic methods `move(dx,dy)`, `draw(graphwin)`, `undraw()`, and `clone()`. Image-specific methods are given below.

`Image(anchorPoint, filename)` Constructs an image from contents of the given file, centered at the given anchor point. Can also be called with `width` and `height` parameters instead of `filename`. In this case, a blank (transparent) image is created of the given width and height.

`getAnchor()` Returns a clone of the point where the image is centered.

`getWidth()` Returns the width of the image.

`getHeight()` Returns the height of the image.

`getPixel(x, y)` Returns a list `[red, green, blue]` of the RGB values of the pixel at position `(x,y)`. Each value is a number in the range 0–255 indicating the intensity of the corresponding RGB color. These numbers can be turned into a color string using the `color_rgb` function (see next section).

Note that pixel position is relative to the image itself, not the window where the image may be drawn. The upper-left corner of the image is always pixel `(0,0)`.

`setPixel(x, y, color)` Sets the pixel at position `(x,y)` to the given color. Note: this is a slow operation.

`save(filename)` Saves the image to a file. The type of the resulting file (e.g., GIF or PPM) is determined by the extension on the filename. For example, `img.save("myPic.ppm")` saves `img` as a PPM file.

6 Generating Colors

Colors are indicated by strings. Most normal colors such as `'red'`, `'purple'`, `'green'`, `'cyan'`, etc. should be available. Many colors come in various shades, such as `'red1'`, `'red2'`,`'red3'`, `'red4'`, which are increasingly darker shades of red.

The graphics module also provides a function for mixing your own colors numerically. The function `color_rgb(red, green, blue)` will return a string representing a color that is a mixture of the intensities of red, green and blue specified. These should be ints in the range 0–255. Thus `color_rgb(255, 0, 0)` is a bright red, while `color_rgb(130, 0, 130)` is a medium magenta.

7 Controlling Display Updates (Advanced)

Usually, the visual display of a `GraphWin` is updated each time the `draw` method is called on an object, or an object's visible state is changed in some way. However, under some circumstances, for example when using the graphics library inside some interactive shells, it may be necessary to *force* the window to update in order for changes to be seen. The `update()` function is provided to do this.

`update()` Causes any pending graphics operations to be carried out and the results displayed.

For efficiency reasons, it is sometimes desirable to turn off the automatic updating of a window every time one of the objects changes. For example, in an animation, you might want to change the appearance of multiple objects before showing the next "frame" of the animation. The `GraphWin` constructor includes a special extra parameter called `autoflush` that controls this automatic updating. By default, `autoflush` is on when a window is created. To turn it off, the `autoflush` parameter should be set to `False`, like this:

```
win = GraphWin("My Animation", 400, 400, autoflush=False)
```

Now changes to the objects in `win` will only be shown when the graphics system has some idle time or when the changes are forced by a call to `update()`.
