# Supplementary material for: An Introduction to Programming for Bioscientists: A Python-Based Primer
Source: PLoS Comput Biol. 2016 Jun 7;12(6):e1004867. doi: 10.1371/journal.pcbi.1004867 (PMC4896647; doi:10.1371/journal.pcbi.1004867)
Supplement: S2 Text — The supplemental text contains sections on: (i) Python as a general language for scientific computing, including the concepts of imperative and declarative languages, Python’s relationship to other languages, and a brief account of languages widely used in the biosciences; (ii) a structured guide to some of the available software packages in computational biology, with an emphasis on Python; and (iii) two sample Supplemental Chapters (one basic, one more advanced), along with a brief, practical introduction to the Python interpreter and integrated development environment (IDE) tools such as IDLE. (PDF) [file pcbi.1004867.s002.pdf]

Supporting Information (S2 Text)

An Introduction to Programming for Bioscientists: A Python-based Primer

Berk Ekmekci◕, Charles E. McAnany◕, Cameron Mura*

Department of Chemistry, University of Virginia, Charlottesville, VA 22904-4319 USA; *cmura@muralab.org; ◕These authors contributed equally to this work.

March 26, 2016

Contents

1 Python in Broader Context: A Tool for Scientific Computing 1

2 An Overview of Bioinformatics Software 3
2.1 Sequence-level Bioinformatics . . . 3
2.2 Structural Bioinformatics . . . 4
2.3 Phylogenetics and Molecular Evolution . . . 5
2.4 Omics-scale Data-processing . . . 6
2.5 Informatics Workflow Management Systems . . . 6
2.6 The Bio* Projects, and Where to Go Next . . . 7

3 Sample Python Chapters 8
3.1 Working with Python: Interpreters, Shells, IDEs . . . 8
3.2 Sample Introductory Topic: Chapter 2 on Variables . . . 8
3.3 Sample Advanced Topic: Chapter 16 on Classes, Objects, OOP . . . 10

References 17

1 Python and the Broader Scientific Computing Ecosystem

Programming languages can be loosely classified, based on various criteria, into distinct lineages or taxonomies. *Imperative* programming languages require the programmer to specify the detailed steps necessary for the program to run, including explicit instructions for modifying data. In an imperative program, the series of statements that comprise the program alter the runtime's state in a predictable and fairly obvious manner; C and Fortran are examples of popular languages that are often used to program in an imperative manner. The Unix shells (`bash`, `csh`, etc.) are also examples of imperative languages: the user types commands one-by-one and the shell executes them in order as it receives them.

In contrast, *declarative* languages emphasize expressions rather than statements. The programmer specifies not the precise steps necessary to generate an answer, but rather expresses the answer directly in terms of the input to the program. For example, in the Prolog programming language, one defines a set of rules (like a system of algebraic equations) and then asks the interpreter if a certain input could satisfy those rules. The interpreter will either find a valid solution or prove that no solutions exist. Regular expressions specify a set of strings and the regex engine tries to match an input string with a given regex. *Functional* languages are declarative programming languages that consider programs to be functions that can be evaluated. As an example, the parallel pipelines in Python (PaPy) toolkit was written in Python, making extensive use of higher-order functions (e.g., `map`, `reduce`), anonymous or 'lambda' functions (see Chapter 13, Functions III), lazy (non-strict) evaluation as its dataflow model, and other elements of functional programming design [1]. Functional languages discourage, and in some cases prohibit, mutation. While `x=x+1` is valid in most imperative languages (the value of `x` is incremented by one), many functional languages have no easy way to change a variable—the programmer would usually refer to `x+1` when that value was needed.

A number of other programming paradigms can be defined, and many of them overlap. Python is considered a *multi-paradigm* language: it provides many of the tools used in functional programming, including a powerful list comprehension syntax, and it also allows the user to define functions as sequences of statements to be executed (the imperative style).

Regardless of the classification scheme, all programs are characterized by two essential features. As mentioned in the main text, these two characteristics are: (i) **algorithms** or, loosely, the 'programming logic', and (ii) **data structures**, or how data are represented/structured, whether they are mutable, etc. [2] Python treats these two features of a program as inseparable, thereby making it particularly well-suited to the object-oriented programming (OOP) paradigm. Indeed, literally everything is an object in Python.

Python has become an especially popular language in scientific computing largely because (i) its clean syntax and straightforward semantics make it a lucid and readily accessible first language in which to learn/extend/maintain code; (ii) as a language, Python is quite expressive [3,4], and is inherently amenable to modern programming paradigms such as OOP and functional programming [5]; (iii) Python's widespread popularity has translated into the development of a rich variety of libraries and third-party toolkits that extend the functionality of the core language into every biological domain, including sequence- and structure-based bioinformatics (e.g., BioPython [6]), comparative genomics (e.g., PyCogent [7]), molecular visualization and modelling toolkits (e.g., PyMOL [8], MMTK [9]), 'omics' data-analysis, data processing pipelines and workflow management systems (e.g., [1]), and even parallel programming [10]. Many of these points are further elucidated in [11].

Several languages other than Python have been widely used in the biosciences; see, e.g., [3] for a comparative analysis. The R programming language provides rich functionality for statistical analysis, and has been widely adopted in bioinformatics (e.g., the Bioconductor project [12]). Perl became an early mainstay in bioinformatics programming (e.g., [13, 14]) largely because of its string-processing capabilities (pattern matching, regular expression handling, etc.). The Fortran, C, and C++ languages offer excellent numerical performance with minimal overhead, making them ubiquitous in computationally-intensive tasks such as molecular dynamics (MD) simulation engines; however, these languages require greater care in memory management and other low-level aspects of writing code, versus higher-level languages such as Python or Perl. The D programming language provides performance near that of C, with many convenient language features for high-level programming; however, the resulting language is complex. Though not a suitable tool for numerical computing, Unix shells (bash, csh, zsh, etc. [15]) are often used to link together other standalone programs (shell scripts, Python code, binary executables, etc.) into ad hoc data-processing pipelines.

2 A Glimpse of the Bioinformatics Software Landscape

There is a vast array of possible options and starting points for software resources in bioinformatics (and, more generally, computational biology), even if we limit our consideration to software that *(i)* is freely distributed under an open-source license and *(ii)* provides low-level libraries or modular toolkits†, rather than feature-complete end-products intended for general purpose usage (including by novices). Monolithic, 'all-in-one' software suites typically have many external *dependencies*, and these dependencies generally correspond to low-level libraries; an example from crystallographic computing is the usage of mmdb, Clipper, and various general-purpose graphics libraries (e.g., OpenGL) to achieve the high-level functionality of the popular Coot molecular graphics program [16].

We can only scratch the surface of available software packages, and the subsections that appear below cover but a handful of the programs often encountered in computational biology. The discussion is intentionally biased towards software written in Python, purely for the pedagogical purposes of this primer. Note that the material which appears below is inherently a moving target (and a fast one, at that). It is not uncommon for scientific software projects and databases to be in various states of flux (see, e.g., the editorial in [17])—new packages appear every few weeks, others disappear or become obsolete, and most software codebases undergo extensive modification on the timescale of months. For these reasons, the material in the following subsections strives to point the reader to various lists and *meta-lists* (lists of lists). Such lists are often more stably persistent (e.g., curated on Wikipedia), and they are inherently able to provide more recently updated catalogs of software than can be provided here. Ultimately, a web-search is often the most effective strategy to discover new information, troubleshoot software, ask programming questions, etc.

The remainder of this section is arranged as subsections based on the following major categories: *(i)* Sequence-level bioinformatics, *(ii)* Structural bioinformatics, *(iii)* Phylogenetics and molecular evolution, *(iv)* Omics-scale data-processing, *(v)* Informatics workflow management systems, and *(vi)* The Bio* projects (and some assorted tips). These categories are the common domains of activity in computational biology, both in terms of software development and practical applications. Within each section we offer pointers to online resources that catalog, in an at least somewhat structured way, some of the codes that exist in that application domain; such information often appears as lists and meta-lists.

2.1 Sequence-level Bioinformatics

This section's content includes: *(i)* pointers to lists of available sequence analysis software packages that are mostly feature-rich, meaning they can be applied as-is to address a production-grade research task; *(ii)* an example of an educational piece of software ('B.A.B.A.') that covers the dynamic programming algorithm, found in many bioinformatics software packages; and *(iii)* practical advice on locating more detailed information and resources, for Python coding and beyond.

- **Lists of software**: An extensive list of sequence alignment codes is at [18]. A wiki is an ideal format for maintaining oft-changing lists of software, as the information can be readily updated by developers, users, and other members of the scientific community. The wiki content cited above is structured by type of application (pairwise sequence alignment, multiple sequence alignment, sequence motif detection, etc.), and a major subsection is dedicated to software for visualization of alignments [19]. Also, a closely related list is at [20], which supplies some programming tools (typically lower-level than the previous two cited URLs) for statistical computing, of the sort that often factors into sequence

†Software can be described as a *low-level library* or *toolkit* if it provides a generic, modular, and reusable set of functions (e.g., a PDB file parser), independent of specific application domains or highly specific instances of tasks (e.g., splitting a PDB file by chain identifier and writing each chain as a separate file); see also §1 of the main text for more discussion of the terms 'low-level' and 'high-level'.

alignment methods. For instance, this latter list describes the software 'Orange' as "*an open source machine learning and data mining software (written in Python). It has a visual programming front-end for explorative data analysis and visualization, and can also be used as a Python library*." Many of the software packages in the above list are open-source, meaning that one can freely access and study the code in order to identify useful chunks of code; these modular units of code can be adapted and re-used for one's own purposes.

- **An educational code: B.A.B.A.**: Though written as a Java applet rather than as Python source code, we mention the 'Basic-Algorithms-of-Bioinformatics Applet' (B.A.B.A.; [21]) because of its pedagogical value in learning the dynamic programming algorithm that underlies many sequence-based methods [22]. Given a user-specified input problem (e.g., two sequence strings to align), the B.A.B.A. applet visually builds the dynamic programming matrix. Users can watch the matrix elements be updated as the algorithm progresses, including for such methods as the Needleman-Wunsch algorithm (globally optimal sequence alignment [23]), the Smith-Waterman method (local sequence alignment [24]) at the heart of the BLAST search method, and the Nussinov algorithm (for prediction of RNA secondary structural regions [25]). Using a tool like B.A.B.A., one can learn the dynamic programming algorithm and play with toy-models in preparation for implementing one's own code in Python.

- **Further resources for coding**: For the hands-on activity of actually implementing an algorithm in Python, the most effective and general path to helpful information is a web search, e.g. using Google. By searching the web, one can discover, for instance, valuable and comprehensive discussions of the 'bottom-up' (exhaustive tabulation) and 'top-down' (recursion, memoization) approaches to dynamic programming (see, e.g., [26], [27], and [28]). This same advice holds true for any algorithm or data structure that one is attempting to implement in Python: websites, and online communities of coders, are invaluable resources for both novice and seasoned programmers.

2.2 Structural Bioinformatics

Both types of software resources for structural bioinformatics—*(i)* feature-rich suites that can be immediately applied to a research task and *(ii)* lower-level Python libraries that are intended more as modules to incorporate into one's own code—can be discovered and used via similar strategies as mentioned above. Namely, we suggest a combination of *(i)* web-search, *(ii)* consulting lists of software on various wikis and other websites (curated sites are particularly helpful), and *(iii)* inspection of existing code from open-source packages. Some more specific notes follow:

- **Structure alignment/analysis**: As an example of a frequent computational task in structural bioinformatics, consider the comparison of two (or more) 3D structures. There are many available packages for optimal pairwise superimposition of two protein structures; the multiple alignment problem is more difficult (and fewer software solutions exist). Many of the available structural alignment packages are tabulated at [29] and, as of this writing, that web resource offers good coverage of existing packages. To visualize the results of structure alignment calculations, one can find numerous possibilities in such lists as [30] and Table 1 of [31].

- **Python-centric suites**: There are many feature-rich, research-grade software suites available for structural analysis tasks (in many cases, these programs also provide advanced visualization capabilities). Several such programs provide a Python API, or a built-in scripting language or shell that resembles Python's syntax. Examples include the Python-based molecular viewing environment PMV [32], the popular PyMOL molecular graphic program [8], and the macromolecular modelling toolkit (MMTK; [9]). PMV supplies extensive functionality for protein structural analysis, with an

emphasis on geometric characteristics (surface curvature, shape properties, etc.). MMTK is "*an open-source program library for molecular simulation applications*", and it provides users with a vast array of Python-based tools. Using MMTK's Python bindings, one can write Python scripts to perform a coarse-grained normal mode calculation for a protein, a simple molecular dynamics (MD) simulation, or molecular surface calculations as part of a broader analysis pipeline.

- **Molecular simulations**: Another type of activity in structural bioinformatics entails molecular modeling and simulation, ranging from simple energy minimization to MD simulations, Monte Carlo sampling, etc. Software packages that are suitable for these purposes are tabulated at [33]. The Bahar lab's 'ProDy' software is an example of a package in this scientific domain that makes substantial use of Python [34]. This "*free and open-source Python package for protein structural dynamics analysis*" is "*designed as a flexible and responsive API suitable for [...] application development*"; this code provides much functionality for principal component analysis and normal mode calculations.
- **Pure Python**: Finally, note that the purely Python-based SciPy toolkit supplies many types of computational geometry utilities that are useful in analyzing macromolecular 3D structures [35]. For instance, the Python module on spatial data structures and algorithms (scipy.spatial [36]) can compute Delaunay triangulations (and, inversely, Voronoi diagrams) and convex hulls of a point-set; this module also supplies data structures, such as kD-trees, that are indispensable in the geometric analysis of proteins and other shapes.

2.3 Phylogenetics and Molecular Evolution

This section describes software resources for computational phylogenetics, a major goal of which is the calculation of phylogenetic trees that accuratey capture the likely evolutionary history of the entities under consideration (be they protein sequences, genes, entire genomes, etc.).

- Wikipedia's list of phylogenetics packages is quite well-developed [37]. Also, a long-time pioneer of the field, J. Felsenstein, maintains a thoroughly curated list of several hundreds of phylogeny-related software packages at [38]. Notably, the software cataloged at this resource can be listed by methodology (general-purpose packages, codes for maximum likelihood methods, Bayesian inference, comparative analysis of trees, etc.); also, that URL provides a list of pointers to other lists. Many of the phylogeny packages listed on the above web-pages are feature-complete and ready for direct application to a research problem (perhaps in a Python script, depending on the package and its API), while others are libraries that serve as sources of lower-level functionality.
- PyCogent: The comparative genomics toolkit, PyCogent, is an example of a Python-based code in the evolutionary genomics domain. This software package supplies "*a fully integrated and thoroughly tested framework for novel probabilistic analyses of biological sequences, devising workflows, etc.*" [7]. As a concrete example of the benefits of the open-source approach, low-level Python functionality for protein 3D structural analysis was added to PyCogent by third-party developers [39], thereby expanding the scope of this (largely sequence-based) code to include structural approaches to molecular phylogenetics.
- DendroPy: A "*Python library for phylogenetic computing*", DendroPy is a codebase that provides "*classes and functions for the simulation, processing, and manipulation of phylogenetic trees and character matrices*". It also "*supports the reading and writing of phylogenetic data in a range of formats, such as NEXUS, NEWICK, NeXML, Phylip, FASTA, etc.*" [40] DendroPy is described by its authors as being able to "*function as a stand-alone library for phylogenetics, a component of more complex multi-library phyloinformatic pipelines, or as a scripting 'glue' that assembles and*

drives such pipelines." This statement perfectly captures the essence of a well-engineered, extensible, open-source scientific software tool, which encourages modularity and code re-use.

- Finally, as an efficient, Python-based approach to developing one's own code in the area of phylogenetics and molecular evolution, the wide-ranging BioPython project (see below) now includes a Bio.Phylo module. This module is described in [41] as supplying "*a unified toolkit for processing, analyzing and visualizing phylogenetic trees in BioPython*."

2.4 Omics-scale Data-processing

The term *omics* refers to the acquisition, analysis, and integration of biological data on a system-wide scale. Such studies have been enabled by the development and application of high-throughput next-generation technologies. Specific sub-fields include, in roughly chronological order of their development, *genomics*, *proteomics*, *metabolomics*, *transcriptomics*, and a panoply of other new omics (*interactomics*, *connectomics*, etc.); the term *NGS* (next-gen sequencing) is closely associated with many of these endeavors. As would be expected, the volume and heterogeneity of data collected on the omics scale present many computing challenges, in terms of both basic algorithms as well as the high-performance software requirements for practical data-processing scenarios. These challenges are largely responsible for spurring the development of many open-source libraries and software packages. Berger et al. [42] recently presented an authoritative review of many computational tools for analyzing omics-scale data, including tables of available software packages sorted by helpful criteria (type of task, type of algorithm). For instance, the review describes potential solutions for data-processing pipelines for transcriptomics data, as obtained from RNA-seq or microarray experiments. An example of an omics-scale software package written chiefly in Python is 'Omics Pipe' [43].

Historically, much of the statistical computing tools that can be used in omics-style bioinformatics has been developed in the R language (see, e.g., the Bioconductor project). A low-level interface between Python and R is available—namely, the RPy package and its recent successor (rpy2) enable the use of R code as a module in Python. As a concrete example of a 'cross-language', integrated omics approach, note that microarray datasets can be processed using established R tools, followed by seamless analysis in Python (via hierarchical clustering) to obtain heat-maps and the corresponding dendrograms [44].

2.5 Informatics Workflow Management Systems

A bioinformatics research project is an inherently computationally-intensive pursuit, often entailing complex workflows of data production or aggregation, statistical processing, and analysis. The data-processing logic, which fundamentally consists of chained transformations of data, can be represented as a *workflow*. Several *workflow management systems* (WMS) have been developed in recent years, in biology and beyond, with the goal of providing robust solutions for data processing, analysis, and provenancing[†]. Available libraries and toolkits enable users to create and execute custom data-processing pipelines (in Python), and feature-rich software frameworks also exist. As mentioned in this section (below), some lightweight, production-grade solutions are written entirely in Python.

WMS software is well-suited to the computational and informatics demands that accompany virtually any major data-processing task. A WMS software suite provides the functional components to enable one to create custom data-processing pipelines, and then deploy (*enact*) these pipelines on either local or distributed compute resources (in the cloud, on an e-science grid, etc.). A WMS can be a high-level, feature-rich, domain-independent software suite (e.g., Taverna [45], KNIME [46]), or a lightweight library that exposes

[†]Loosely, *data provenance* involves carefully logging results (and errors, exceptions, etc.), ensuring reproducibility of workflows by automatically recording run-time parameters, and so on.

modular software components to users (e.g., PaPy [1]). Usage of a WMS is roughly similar in spirit to using, say, a series of low-level Unix scripts as wrappers for data-processing tools; however, compared to the one-off scripting approach, most WMS solutions feature greater flexibility and extensibility, enhanced robustness, and are generally applicable in more than one scientific domain. WMS suites that are often employed in bioinformatics are described, including literature references and software reviews, in an article that reports the creation of a lightweight Python library known as PaPy [1]. PaPy is a purely Python-based tool that enables users to create and execute modular data-processing pipelines, using the functional programming and flow-based programming paradigms. Alongside the PaPy toolkit, other Python-based WMS solutions include Ruffus [47] and Omics Pipe [43].

2.6 The Bio* Projects, and Where to Go Next

This final section lists the many available ‘Bio*’ projects, where the ‘*’ wildcard is a placeholder for a particular programming language; typically, the language at the core of a given Bio* project has seen sufficiently widespread usage in computational biology to warrant the concerted development of general-purpose libraries in that language; notable examples are the the BioPython and BioPerl projects. The table below provides a sampling of these projects, which are cataloged more thoroughly at the Open Bioinformatics Foundation [48]. The table is followed by a few assorted tips and pointers to aid in the discovery of additional resources.

Table S1: The Bio* projects

**Project**	**Description** (from each respective website)
**BioPython** [6]	"Biopython is a set of freely available tools for biological computation written in Python by an international team of developers. It is a distributed collaborative effort to develop Python libraries and applications which address the needs of current and future work in bioinformatics."
**BioPerl** [13]	"a community effort to produce Perl code which is useful in biology"
**BioJava** [49]	"BioJava is an open-source project dedicated to providing a Java framework for processing biological data. It provides analytical and statistical routines, parsers for common file formats and allows the manipulation of sequences and 3D structures. The goal of the biojava project is to facilitate rapid application development for bioinformatics."
**BioSQL** [50]	"BioSQL is a generic relational model covering sequences, features, sequence and feature annotation, a reference taxonomy, and ontologies (or controlled vocabularies)."
**BioRuby** [51]	"Open source bioinformatics library for Ruby"

We conclude by noting the following potentially useful resources:

- Wikipedia’s top-level page on bioinformatics software, organized by categories, is at [52]. At a similar level of detail, there exist open-access journals that will be of interest (e.g., *Algorithms for Molecular Biology*); also, *PLOS Computational Biology* publishes new contributions in a dedicated ‘Software Collection’ [53].
- The bioinformatics.org site [54] includes a list of hundreds of packages for bioinformatics software development; brief annotations for many of these packages are available at a related URL [55]. In addition, ref [56] lists software for Linux, sorted by categories. One can search for ‘python’ on that page, and will find numerous codes of potential interest.
- Another useful online search strategy is to query PyPI, the Python Package Index [57]. Searching PyPI with terms such as ‘bioinformatics’ will retrieve numerous potentially useful hits (a beneficial feature of this approach is that the returned codes are likely to be under active, or at least recent, development).

3 Supplemental Chapters of Python Code: Two Samples

In addition to the examples of Python code in the main text, a suite of Supplemental Chapters is provided with this work. These freely-available Chapters cover the topics enumerated in Table 1 (main text), and the latest source files are maintained at `http://p4b.muralab.org`. Each Chapter is written as a Python file—i.e., each Chapter is a plaintext `.py` file that can be read, executed, modified, etc. as would any ordinary Python source code. For pedagogical purposes, each Chapter is heavily annotated with explanatory material. These explanations take the form of comments in the Python code (lines that begin with a pound sign, '`#`'), thereby allowing the Chapters to be both descriptive and executable. The remainder of this section consists of two sample chapters, following a brief subsection that describes some practicalities of interacting with Python in a Unix shell environment. (Python is cross-platform, and various Python interpreters are freely available on the Windows and Apple OS X operating systems too.)

3.1 The Python Interpreter, the Unix Shell, and IDEs

Virtually all modern Linux distributions include a recent version of Python. One can begin an interactive Python session by accessing the *interpreter* (see §1 of the main text for more on this term). This, in turn, can be achieved by opening a Unix shell (terminal) and typing the standard command `python`. (On systems with multiple versions of Python installed [not an uncommon scenario], the command `python3` may be necessary—one should experiment with this on one's own Linux system.) As a concrete example, if the first Supplemental Chapter on control flow (the file ch05controlFlowI.py) is present in the current working directory, then its contents can be imported into an interactive Python session by issuing the statement `import ch05controlFlowI` (note the missing '`.py`' suffix) at the Python prompt. The default Python prompt is indicated by three greater-than signs (>>>); for line continuation, the prompt appears as three periods (`...`). There are alternatives to the default Python interpreter, such as the freely-available IPython command shell [58]. In IPython, one can 'load' this file by typing either `import ch05controlFlowI` (as above) or else `run ch05controlFlowI.py` (note the file extension). Another Python interpreter (and source code editor) is IDLE. This integrated development environment (IDE) for Python is fairly straightforward to use, and IDLE is bundled with all standard, freely available distributions of Python. IDLE provides an interactive session and a simple file editor for creating Python source code. The Supplemental Chapters are simple text files that can be loaded into the IDLE editor and executed. Beyond the popular IPython and IDLE, other options for Python IDEs also exist. For instance, many users on the Windows OS use the Anaconda Python distribution, which is packaged with a large number of scientific computing extensions already pre-configured [59].

3.2 A Sample Chapter at a Basic Level: Variables [Chap 2]

The following code is Supplemental Chapter `ch02variables.py`, which is an introductory-level treatment of variables.

```
"""Programming for Biochemists, lessons for Python programming.
Copyright (C) 2013, Charles McAnany. This program is free software: you can
redistribute it and/or modify it under the terms of the GNU Affero General
Public License as published by the Free Software Foundation, either version 3
of the License, or (at your option) any later version. This program is
distributed in the hope that it will be useful, but WITHOUT ANY WARRANTY;
without even the implied warranty of MERCHANTABILITY or FITNESS FOR A
PARTICULAR PURPOSE. See the GNU Affero General Public License for more details.
You should have received a copy of the GNU Affero General Public License along
with this program. If not, see <http://www.gnu.org/licenses/>."""
#Chapter 2: Using variables.
#We can tell Python to remember the result of a calculation, by storing it
#in a variable. The syntax is
```

```
#variableName = calculation
#Here, let me make a variable to store seven times seven.
def sevSqu():
    sevenSquared = 7 * 7
    # I can now use sevenSquared anywhere in this function.
    print(sevenSquared)

#Rules for variables:
#Variables must start with a letter or underscore. After that, any combination
#of numbers and letters is OK. These are some good variable names:
# value1
# fooBar
# juice_concentration_2
#But these are not valid variables.
# 1stValue (starts with a number)
# let me in (has spaces in it.)
# I1lI1l (technically valid, but I'll kill you if you use this.)

##### NOTA BENE:
#Variables in python are case sensitive!
# aNumber is a totally different variable than Anumber. This can lead to very
#subtle bugs. So:
# Use consistent naming schemes. I will almost always capitalize the first
# letter of each word in my variables, except the first one. My variables
# might be:
# aLittleBit
# numberOfRottenBananas
# counter (only one word, so no capitals.)
# (programmers refer to this capitalization scheme as camel casing.)


#Some variable use:
def onePlusMe():
    number = 5
    number = number + 1
    print(number)
# Hm?! Okay, this deserves an explanation.
#When python sees this, here's what it will do.
# 1. it sees that you're assigning a variable.
# 2. it calculates the value you're going to assign. Here, it's number + 1
# 3. it sets number to that value.

#So remember, = is NOT a question. It's an instruction.
#In a mathematics course, if you were told that x=x+1, your initial
#response might be along the lines of "no it isn't", and rightly so!
#In Python, it means "Calculate number + 1. Number is now that value."

#Here's a program to convert temperatures from Celsius to Fahrenheit.
def celsToFahr():
    celsiusTemperature = 30
    fahrenheitTemperature = 9/5 * celsiusTemperature + 32
    # Python does order of operations correctly.
    print("the temperature is ", fahrenheitTemperature, "degrees Fahrenheit.")
# Print is a very strange function, in that it can take many arguments
# (separated by commas). For now, know that it will glue the arguments
# together and print the whole thing.

#For some of the programs you'll write, you need to look at the characters
#in strings. I'll cover the syntax in greater detail is CH08, collections I,
#but for now I'll just give you some useful syntax for strings:
#To read the kth character of a string, you say stringName[k-1]
#Most of the time, you'll just need the first character, which we can
#extract with stringName[0]
def stringManip():
    initialString = "aoeu"
    print("the string starts with ", initialString[0])
    #We could get the third character like this:
    print("The third character is ", initialString[2])
```

```
    #It's 2, not 3, because we use k-1 for the number, not k.
    #(the reason becomes much clearer in CH08)

#Good? Good.


###################
##   Exercises   ##
###################
# 1. Rewrite the temperature program to convert a Fahrenheit temperature to
# a celsius one. What is the celsius temperature when it is 100 F?
# Reminder: Celsius = 5/9 (Fahrenheit - 32)
def fahrToCels():
    pass #again, delete the pass, replace this function with your code.



#2. Track the value of each of the following variables during this program.
#Just fill out the table with the values as they change.
#(don't run the code, do it by hand.)
def exercise2():
                        # a | b | c #
    a = 1               # 1 | ? | ? #
    b = 1               # 1 | 1 | ? #
    c = 1               # 1 | 1 | 1 #
    a = b + c           # 2 | 1 | 1 #
    b = a + c           # 2 |   |   #
    c = b + a           #   |   |   #
    b = c               #   |   | 5 #
    a = a + b           #   |   |   #
    c = c * c           # 7 | 5 |   #

#3. Print the first three characters of the specified string:
def printChars():
    someChars = "aoeuhtns"
```

3.3 A Sample Chapter at a More Advanced Level: Classes & Objects, II [Chap 16]

The following code is Supplemental Chapter `ch16ClassesObjectsII.py`, which is a more advanced presentation of classes, objects, and object-oriented programming.

```
"""Programming for Biochemists, lessons for Python programming.
Copyright (C) 2013, Charles McAnany. This program is free software: you can
redistribute it and/or modify it under the terms of the GNU Affero General
Public License as published by the Free Software Foundation, either version 3
of the License, or (at your option) any later version. This program is
distributed in the hope that it will be useful, but WITHOUT ANY WARRANTY;
without even the implied warranty of MERCHANTABILITY or FITNESS FOR A
PARTICULAR PURPOSE. See the GNU Affero General Public License for more details.
You should have received a copy of the GNU Affero General Public License along
with this program. If not, see <http://www.gnu.org/licenses/>."""
#Chapter 16: Objects and classes: philosophy and iterators.
#This is it, the penultimate chapter! You will use everything you've learned
#up to this point in this chapter. It should be fun!

#I'm going to start this chapter by giving you something practical:
#an example of a fully-formed class that shows you how classes
#are used. A rational number is one of the form a/b, where a and b are
#integers. Python does not have built-in support for rationals
#and rational arithmetic.
class RationalNumber:
    """A class that implements a rational number and the necessary
    Arithmetic operations on it."""
    def __init__(self,numerator, denominator):
        """Arguments should be numbers or RationalNumbers, and will
        be the values of this rational number's numerator and denominator."""
```

```
        if(isinstance(numerator, RationalNumber)):
            if(isinstance(denominator, RationalNumber)):
               #The constructor was called with RationalNumbers
               self._n = numerator._n * denominator._d
               self._d = denominator._n * numerator._d
            else:
                #The numerator, but not denominator, is a RationalNumber
                self._n = numerator._n
                self._d = denominator* numerator._d
        else:
            if(isinstance(denominator, RationalNumber)):
               #The denominator, but not numerator, is a RationalNumber
               self._n = numerator * denominator._d
               self._d = denominator._n
            else:
                #Both arguments are plain old numbers
                self._n = numerator
                self._d = denominator
        if(self._n != 0):
            self.reduceFraction()
        else:
            self._d = 1

    def reduceFraction(self):
        gcd = greatestDivisor(self._n, self._d)
        self._n //= gcd
        self._d //= gcd

    def add(self, otherNum):
        """Adds a rational number to this one, using the fact that
        a/b + c/d = (a*d + c*b)/(b*d)"""
        return RationalNumber(self._n*otherNum._d+otherNum._n*self._d, self._d*otherNum._d)

    def subtract(self,otherNum):
        negOther = RationalNumber(-otherNum._n, otherNum._d)
        return self.add(negOther)

    def mult(self, otherNum):
        return RationalNumber(self._n * otherNum._n, self._d * otherNum._d)

    def divide(self, otherNum):
        return RationalNumber(self._n * otherNum._d, self._d * otherNum._n)
    def __str__(self):
        return "{0:d}/{1:d}".format(self._n, self._d)

#I put the code for GCD outside the class - it's not really associated with
#rational numbers, so it should be in a different place.
def greatestDivisor(a,b):
    if(b == 0):
        return a
    return greatestDivisor(b,a % b)

def useRational():
    #a = 1/2
    a = RationalNumber(1,2)
    #b = 1/3
    b = RationalNumber(1,3)
    #c = a + b
    c = a.add(b)
    print(c)
    #Now to demonstrate that rationals are truly precise...
    storage = RationalNumber(0,1)
    floatSum = 0
    for i in range(1000):
        storage = storage.add(RationalNumber(1,1000))
        floatSum += 0.001
    print(floatSum)
    print(storage)
```

```
    floatZero = floatSum - 1.0
    storageZero = storage.subtract(RationalNumber(1,1))
    print(floatZero)
    print(storageZero)
    #The floating point version has some noise that has accumulated during
    #the computation. The rational does not have this noise.



#Next: Something practical. You know how you can do
#for i in range(10):
#, right? Well, range is just a class with a few methods defined.
#A class is iterable (may be used with a for loop) if it defines the
#method __iter__() that returns an object with a method called __next__().
#__next__() should return the next value in the sequence or raise
#a StopIteration exception.

class NewRange():
    def __init__(self, start, stop):
        print("NewRange.__init__")
        self._start = start
        self._stop = stop
    def __iter__(self):
        print("NewRange.__iter__")
        return RangeIterator(self._start,self._stop)

class RangeIterator():
    def __init__(self,start,stop):
        print("RangeIterator.__init__")
        self._currPos = start
        self._endPos = stop
    def __next__(self):
        print("RangeIterator.__next__", end = " ")
        if self._currPos < self._endPos:
            self._currPos = self._currPos + 1
            print(" -> {0:d}".format(self._curPos-1))
            return self._currPos - 1 #-1 because I already incremented, return
        else:                        #what the value was, not what it is.
            print(" -> StopIteration")
            raise StopIteration


#If your class contains a method called __next__(), you can have __iter__
#just return self:

class SimpleRange:
    def __init__(self,start,stop):
        self._currPos = start
        self._endPos = stop
    def __next__(self):
        if self._currPos < self._endPos:
            self._currPos = self._currPos + 1
            return self._currPos - 1 #-1 because I already incremented, return
        else:                        #what the value was, not what it is.
            raise StopIteration
    def __iter__(self):
        return self

#When Python comes to a for loop, it first calls __iter__(), then repeatedly
#calls __next__() on that iterator until it throws StopIteration.
#The advantage is we can just use it like a normal range.
def useNewRange():
    nr = NewRange(0,10)
    for i in nr:
        print (i)
    sr = SimpleRange(0,10)
    for i in sr:
        print(i)
```

```


#Okay, let's get biochemical again. Consider a class that stores DNA:
class DNAStore:
    """Represents a strand of DNA. Accepts new dna as strings or collections
    of strings. """
    _bases = "" #Currently empty.

    def __init__(self, bases):
        """bases is a string or a sequence of strings that will be added to
        this objects' dna store."""
        self.add(bases)
        print("Initialized DNA strand with {0:s}".format(self._bases))

    def add(self, newDNA):
        """Adds new dna to the end of this strand. Rules for dna are the same
        as for the initializer."""
        if isinstance(newDNA, str):
            for base in newDNA:
                self._addLetter(base)
        elif isinstance(newDNA, (tuple,list)):
            for thing in newDNA:
                self.add(thing) #If it's a tuple or list, split it and add
                                 #each part of it recursively.
        else:
            raise Exception("Invalid DNA.")

    def _addLetter(self, base):
        if base in "AGTC":
            self._bases = self._bases + base
        else:
            raise Exception("Unknown letter for DNA: {0:s}".format(base))

    def getBases(self):
        return self._bases


#I'd like to extend this class to allow me to iterate over the codons.
class IterableDNA(DNAStore):
    """An iterable version of a DNA store. Iterates by *codon*, not by
    *base*."""
    _bases = "" #Currently empty.

    def __init__(self, bases):
        """bases is a string or a sequence of strings that will be added to
        this objects' dna store."""
        self.add(bases)
        print("Initialized DNA strand with {0:s}".format(self._bases))

    def add(self, newDNA):
        """Adds new dna to the end of this strand. Rules for dna are the same
        as for the initializer."""
        if isinstance(newDNA, str):
            for base in newDNA:
                self._addLetter(base)
        elif isinstance(newDNA, (tuple,list)):
            for thing in newDNA:
                self.add(thing) #If it's a tuple or list, split it and add
                                 #each part of it recursively.
        else:
            raise Exception("Invalid DNA.")

    def _addLetter(self, base):
        if base in "AGTC":
            self._bases = self._bases + base
        else:
            raise Exception("Unknown letter for DNA: {0:s}".format(base))

```

```
    def getBases(self):
        return self._bases

    def __iter__(self):
        #Initialize the iteration.
        self._iterPos = 0
        return self
    def __next__(self):
        start = self._iterPos
        self._iterPos = start + 3
        if(len(self._bases) - start < 3):
            raise StopIteration
        codon = self._bases[start:start + 3]
        return codon

def iterateDNA():
    idna = IterableDNA("AGTGACTAGTCACTACTAGCATGAGACATGACGAT")
    for cdn in idna:
        print(cdn)
        #The big point here is that the person using your class needn't
        #think about how the iteration works; it "just works" and is clear
        #and simple.

###################
##   Exercises   ##
###################
#1. Add a method to DNAStore that calculates the GC content of its stored
#dna.

#2. Add a method to DNAStore that accepts another DNAStore, and calculates
#the Hamming distance between itself and the other strand.

# 3.Explain the behavior of this function:
def rangeMess():
    def printNest(iterable):
        for i in iterable:
            for j in iterable:
                print("i = {0}, j = {1}.".format(i,j))

    a = range(0,10)
    b = NewRange(0,10)
    c = SimpleRange(0,10)
    print("built-in range:")
    printNest(a)
    print("NewRange:")
    printNest(b)
    print("SimpleRange:")
    printNest(c)

#4. If you play with IterableDNA, you'll notice it has the behavior of
#SimpleRange: You can't nest iteration. Fix it.
class BetterIterableDNA:
    pass

#5. Implement a deque class. (See CH12, circles() for a brief discussion of
#deques.)
#It should support these operations
#pushLeft(thing) :: appends thing to the left end of the deque.
#popLeft()       :: removes the leftmost item from the deque.
#peekLeft()      :: returns the leftmost item from the deque.
#and their corresponding right-side methods.
class Deque:
    pass

#I have provided this test method for your use:
def testDeque():
    def checkEqual(a,b):
        if (a != b):
```

```
            raise Exception("unequal: {0}, {1}".format(a,b))
    def checkBroken(op):
        """Tries to run op (which should be a zero-argument function). If op raises
    an exception, this catches it and returns gracefully. If op does *not* raise
    an exception, this raises its own to indicate that the code did not fail."""
        try:
            op()
        except(Exception):
            print("Error occured as expected.")
            return
        raise Exception("Code did not indicate an error.")
                                    # D1            D2
    d1 = Deque()                    # <>
    d1.pushLeft(1)                  # <1>
    d1.pushRight(2)                 # <1, 2>
    checkEqual(d1.peekLeft(), 1)    # <1, 2>
    checkEqual(d1.peekLeft(), 1)    # <1, 2>
    d1.popLeft()                    # <2>
    checkEqual(d1.peekLeft(), 2)    # <2>
    #Can the class support being emptied?
    d1.popRight()                   # <>
    #Does the class support strange objects being inserted?
    d1.pushRight((3,4))             # <(3,4)>
    d1.pushLeft("aoeu")             # <"aoeu", (3,4)>
    checkEqual(d1.peekRight(), (3,4))#<"aoeu", (3,4)>
    d2 = Deque()                    #   '           <>
    d2.pushLeft(2)                  #   '           <2>
    #Are multiple objects truly independent?
    checkEqual(d2.peekRight(), 2)   #   '           <2>
    d1.popLeft()                    #  <(3,4)>      <2>
    d1.popLeft()                    #  <>           <2>
    #Beat up the class a bit...
    for i in range(10000):
        d1.pushLeft(i)              # <10000, 9999, ... 1, 0>
    for i in range(5000):
        d1.popRight()               #<10000, 9999, ... 5001, 5000>
    checkEqual(d1.peekRight(), 5000)

    d3 = Deque()
    #Does it indicate a problem if I try to remove or read from an empty deque?
    checkBroken(lambda:d3.popRight())
    checkBroken(lambda:d3.peekLeft())
    #Does the deque still work correctly after I try to manipulate it when
    #empty?
    d3.pushLeft(1)
    checkEqual(d3.peekRight(),1)

#6. Make your deque class iterable. The iteration should start at the left and
#yield all the elements, just like for a list. Iterating should NOT destroy
#the deque being used. That is, after I iterate it, I should be able to push
#and pop and peek just as before and all the values must be the same. As an
#example, the following __next__() would violate this requirement:
#def __next__(self):
#    if(self._isEmpty()):
#         raise StopIteration
#    self.popLeft()
#    return self.peekLeft()
#(Assuming, of course, that self refers to the original deque)

#(If you implemented your deque well, this should not be hard!) Note: You may
#assume that the deque is not modified during the iteration, so, for example,
#the behavior of the following code is undefined, and will not be tested:
#   for elem in deq:
#       deq.popRight() #Undefined behavior: Deque is modified during iteration.
#       print(elem)
#       elem = elem+1  #Also undefined: I'm trying to modify the elements.
#You can assume that the iterator will not be nested; if it works like
#SimpleRange, that's okay.
```

```
class IterableDeque(Deque):
    pass

#7.
#Write a method to stress-test your deque, like the tests above.
def testIterableDeque():
    pass
```

References

1. Cieślik M, Mura C. A Lightweight, Flow-based Toolkit for Parallel and Distributed Bioinformatics Pipelines. BMC Bioinformatics. 2011;12:61. Available from: http://dx.doi.org/10.1186/1471-2105-12-61.

2. Wirth N. Algorithms + Data Structures = Programs. Prentice-Hall Series in Automatic Computation. Prentice Hall; 1976.

3. Fourment M, Gillings MR. A Comparison of Common Programming Languages Used in Bioinformatics. BMC Bioinformatics. 2008;9(1):82. Available from: http://dx.doi.org/10.1186/1471-2105-9-82.

4. Evans D. Introduction to Computing: Explorations in Language, Logic, and Machines. CreateSpace Independent Publishing Platform; 2011. Available from: http://www.computingbook.org.

5. Hinsen K. The Promises of Functional Programming. Comput Sci Eng. 2009 Jul;11(4):86–90. Available from: http://dx.doi.org/10.1109/MCSE.2009.129.

6. Cock PJA, Antao T, Chang JT, Chapman BA, Cox CJ, Dalke A, et al. BioPython: Freely Available Python Tools for Computational Molecular Biology and Bioinformatics. Bioinformatics. 2009 Mar;25(11):1422–1423. Available from: http://dx.doi.org/10.1093/bioinformatics/btp163.

7. Knight R, Maxwell P, Birmingham A, Carnes J, Caporaso JG, Easton BC, et al. PyCogent: A toolkit for making sense from sequence. Genome Biology. 2007;8(8):R171. Available from: http://dx.doi.org/10.1186/gb-2007-8-8-r171.

8. The PyMOL Molecular Graphics System, Schrödinger, LLC;. Available from: http://pymol.org.

9. Hinsen K. The Molecular Modeling Toolkit: A New Approach to Molecular Simulations. Journal of Computational Chemistry. 2000 Jan;21(2):79–85. Available from: http://dx.doi.org/10.1002/(SICI)1096-987X(20000130)21:2<79::AID-JCC1>3.0.CO;2-B.

10. Hinsen K, Langtangen HP, Skavhaug O, Åsmund Ødegård. Using BSP and Python to simplify parallel programming. Future Generation Computer Systems. 2006;22(1–2):123 – 157. Available from: http://www.sciencedirect.com/science/article/pii/S0167739X03002061.

11. Hinsen K. High-Level Scientific Programming with Python. In: Proceedings of the International Conference on Computational Science-Part III. ICCS '02. London, UK, UK: Springer-Verlag; 2002. p. 691–700.

12. Gentleman RC, Carey VJ, Bates DM, Bolstad B, Dettling M, Dudoit S, et al. Bioconductor: Open Software Development for Computational Biology and Bioinformatics. Genome Biology. 2004;5(10):R80. Available from: http://dx.doi.org/10.1186/gb-2004-5-10-r80.

13. Stajich JE, Block D, Boulez K, Brenner SE, Chervitz SA, Dagdigian C, et al. The BioPerl Toolkit: Perl Modules for the Life Sciences. Genome Research. 2002 Oct;12(10):1611–1618. Available from: http://dx.doi.org/10.1101/gr.361602.

14. Tisdall JD. Mastering Perl for Bioinformatics. O'Reilly Media; 2003.

15. Robbins A, Beebe NHF. Classic Shell Scripting: Hidden Commands that Unlock the Power of Unix. O'Reilly Media; 2005.

16. Emsley P, Lohkamp B, Scott WG, Cowtan K. Features and Development of Coot. Acta Crystallographica Section D—Biological Crystallography. 2010;66:486–501.

17. Wren JD, Bateman A. Databases, Data Tombs and Dust in the Wind. Bioinformatics. 2008 Sep;24(19):2127–2128. Available from: http://dx.doi.org/10.1093/bioinformatics/btn464.

18. Wikipedia. List of Sequence Alignment Software; 2016. Available from: http://en.wikipedia.org/wiki/List_of_sequence_alignment_software.

19. Wikipedia. List of Alignment Visualization Software; 2016. Available from: http://en.wikipedia.org/wiki/List_of_alignment_visualization_software.

20. Wikipedia. List of Statistical Packages; 2016. Available from: http://en.wikipedia.org/wiki/List_of_statistical_packages.

21. Casagrande N. Basic-Algorithms-of-Bioinformatics Applet; 2015. Available from: http://baba.sourceforge.net.

22. Eddy SR. What is Dynamic Programming? Nat Biotechnol. 2004 Jul;22(7):909–910.

23. Needleman SB, Wunsch CD. A general method applicable to the search for similarities in the amino acid sequence of two proteins. J Mol Biol. 1970 Mar;48(3):443–453.

24. Smith TF, Waterman MS. Identification of common molecular subsequences. J Mol Biol. 1981 Mar;147(1):195–197.

25. Nussinov R, Jacobson AB. Fast algorithm for predicting the secondary structure of single-stranded RNA. Proc Natl Acad Sci USA. 1980 Nov;77(11):6309–6313.

26. Guest. Dynamic programming and memoization: bottom-up vs top-down approaches; 2011. Available from: http://stackoverflow.com/questions/6164629/dynamic-programming-and-memoization-bottom-up-vs-top-down-approaches.

27. Voithos. Dynamic programming solution to knapsack problem; 2016. Available from: http://codereview.stackexchange.com/questions/20569/dynamic-programming-solution-to-knapsack-problem.

28. Miller B, Ranum D. Dynamic Programming—Problem Solving with Algorithms and Data Structures; 2014. Available from: http://interactivepython.org/runestone/static/pythonds/Recursion/DynamicProgramming.html.

29. Wikipedia. Structural Alignment Software; 2015. Available from: http://en.wikipedia.org/wiki/Structural_alignment_software.

30. Wikipedia. List of Molecular Graphics Systems; 2016. Available from: http://en.wikipedia.org/wiki/List_of_molecular_graphics_systems.

31. O'Donoghue SI, Goodsell DS, Frangakis AS, Jossinet F, Laskowski RA, Nilges M, et al. Visualization of Macromolecular Structures. Nature Methods. 2010 Mar;7(3s):S42–S55. Available from: http://dx.doi.org/10.1038/nmeth.1427.

32. Sanner M. Python: a programming language for software integration and development. J Mol Graphics Mod. 1999;17:57–61.

33. Wikipedia. List of Software for Molecular Mechanics Modeling; 2016. Available from: http://en.wikipedia.org/wiki/List_of_software_for_molecular_mechanics_modeling.

34. Bakan A, Meireles LM, Bahar I. ProDy: Protein Dynamics Inferred from Theory and Experiments. Bioinformatics. 2011;27(11):1575–1577. Available from: http://bioinformatics.oxfordjournals.org/content/27/11/1575.abstract.

35. Jones E, Oliphant T, Peterson P, et al.. SciPy: Open-source Scientific Tools for Python; 2001–. [Online; accessed 2015-06-30]. Available from: http://www.scipy.org/.

36. Scipy community. Spatial data structures and algorithms; 2016. Available from: http://scipy.github.io/devdocs/tutorial/spatial.html.

37. Wikipedia. List of Phylogenetics Software; 2015. Available from: http://en.wikipedia.org/wiki/List_of_phylogenetics_software.

38. Felsenstein J. Phylogeny Programs; 2014. Available from: http://evolution.genetics.washington.edu/phylip/software.html.

39. Cieślik M, Derewenda ZS, Mura C. Abstractions, Algorithms and Data Structures for Structural Bioinformatics in PyCogent. Journal of Applied Crystallography. 2011 Feb;44(2):424–428. Available from: http://dx.doi.org/10.1107/S0021889811004481.

40. Sukumaran J, Holder MT. DendroPy: a Python library for phylogenetic computing. Bioinformatics. 2010 Jun;26(12):1569–1571.

41. Talevich E, Invergo BM, Cock PJ, Chapman BA. Bio.Phylo: A unified toolkit for processing, analyzing and visualizing phylogenetic trees in Biopython. BMC Bioinformatics. 2012;13(1):1–9. Available from: http://dx.doi.org/10.1186/1471-2105-13-209.

42. Berger B, Peng J, Singh M. Computational Solutions for Omics Data. Nature Reviews, Genetics. 2013 Apr;14(5):333–346. Available from: http://dx.doi.org/10.1038/nrg3433.

43. Fisch KM, Meissner T, Gioia L, Ducom JC, Carland TM, Loguercio S, et al. Omics Pipe: a community-based framework for reproducible multi-omics data analysis. Bioinformatics. 2015 Jun;31(11):1724–1728.

44. Cock P. Using Python (and R) to draw a Heatmap from Microarray Data; 2010. Available from: http://www2.warwick.ac.uk/fac/sci/moac/people/students/peter_cock/python/heatmap.

45. Oinn T, Addis M, Ferris J, Marvin D, Senger M, Greenwood M, et al. Taverna: A Tool for the Composition and Enactment of Bioinformatics Workflows. Bioinformatics. 2004 Jun;20(17):3045–3054. Available from: http://dx.doi.org/10.1093/bioinformatics/bth361.

46. Berthold MR, Cebron N, Dill F, Gabriel TR, Kötter T, Meinl T, et al. KNIME - The Konstanz Information Miner. SIGKDD Explorations. 2009;11(1).

47. Goodstadt L. Ruffus: A Lightweight Python Library for Computational Pipelines. Bioinformatics. 2010 Nov;26(21):2778–2779.

48. Open Bioinformatics Foundation. Projects; 2015. Available from: http://www.open-bio.org/wiki/Projects.

49. Pocock M, Down T, Hubbard T. BioJava: Open Source Components for Bioinformatics. SIGBIO Newsl. 2000 Aug;20(2):10–12. Available from: http://doi.acm.org/10.1145/360262.360266.

50. Main Page - BioSQL;. Available from: http://www.biosql.org/wiki/Main_Page.

51. Goto N, Prins P, Nakao M, Bonnal R, Aerts J, Katayama T. BioRuby: Bioinformatics Software for the Ruby Programming Language. Bioinformatics. 2010 Oct;26(20):2617–2619.

52. Wikipedia. Category:Bioinformatics software; 2014. Available from: http://en.wikipedia.org/wiki/Category:Bioinformatics_software.

53. Prlić A, Lapp H. The PLOS Computational Biology Software Section. PLOS Computational Biology. 2012 Nov;8(11):e1002799. Available from: http://dx.doi.org/10.1371/journal.pcbi.1002799.

54. Bioinformatics org. Bioinformatics.org; 2016. Available from: http://www.bioinformatics.org.

55. Bioinformatics org. Software Map; 2016. Available from: http://www.bioinformatics.org/groups/categories.php?cat_id=2a.

56. Sicheritz-Ponten T. Molecular Biology related programs for Linux; 2016. Available from: http://www.bioinformatics.org/software/mol_linux_cat.php3.

57. PyPI: The Python Package Index;. Available from: http://pypi.python.org.

58. Pérez F, Granger BE. IPython: a System for Interactive Scientific Computing. Computing in Science and Engineering. 2007 May;9(3):21–29. Available from: http://ipython.org.

59. Anaconda;. Available from: https://www.continuum.io/.
